# Benefit of minimally invasive extracorporeal circulation on minimally invasive aortic valve replacement through right lateral mini-thoracotomy using femoral cannulation: a propensity-matched analysis

**DOI:** 10.1093/icvts/ivae224

**Published:** 2024-12-30

**Authors:** Yoshitsugu Nakamura, Taisuke Nakayama, Kusumi Niitsuma, Yuka Higuma, Masaki Ushijima, Miho Kuroda, Yuto Yasumoto, Yujiro Ito, Yujiro Hayashi, Ryo Tsuruta, Naoya Yamauchi, Akihiro Higashino, Fumiaki Shikata

**Affiliations:** Department of Cardiovascular Surgery, Chibanishi General Hospital, Chiba, Japan; Department of Cardiovascular Surgery, Chibanishi General Hospital, Chiba, Japan; Department of Cardiovascular Surgery, Chibanishi General Hospital, Chiba, Japan; Department of Cardiovascular Surgery, Chibanishi General Hospital, Chiba, Japan; Department of Cardiovascular Surgery, Chibanishi General Hospital, Chiba, Japan; Department of Cardiovascular Surgery, Chibanishi General Hospital, Chiba, Japan; Department of Cardiovascular Surgery, Chibanishi General Hospital, Chiba, Japan; Department of Cardiovascular Surgery, Chibanishi General Hospital, Chiba, Japan; Department of Cardiovascular Surgery, Chibanishi General Hospital, Chiba, Japan; Department of Cardiovascular Surgery, Chibanishi General Hospital, Chiba, Japan; Department of Medical Engineering, Chibanishi General Hospital, Chiba, Japan; Department of Cardiovascular Surgery, Chibanishi General Hospital, Chiba, Japan; Department of Cardiovascular Surgery, Kitasato University Hospital, Sagamihara, Kanagawa, Japan

**Keywords:** extracorporeal circulation, minimally invasive extracorporeal circulation, minimally invasive aortic valve replacement, blood transfusion, cerebral infarction, asymptomatic brain injury

## Abstract

**OBJECTIVES:**

The objective of this study was to evaluate the impact of minimally invasive extracorporeal circulation on blood transfusion and asymptomatic brain injury in comparison to conventional extracorporeal circulation in the context of minimally invasive aortic valve replacement through right lateral mini-thoracotomy surgery.

**METHODS:**

This was a retrospective observational study. Patients who underwent isolated aortic valve replacement through right lateral mini-thoracotomy surgery were divided into two groups: the minimally invasive extracorporeal circulation group and the conventional extracorporeal circulation group. Propensity matching was employed for further analysis.

**RESULTS:**

Of 242 patients, the minimally invasive group and conventional group comprised 166 patients and 76 patients, respectively. In the matched cohort of 71 pairs, the two groups had similar preoperative characteristics. Extracorporeal circulation time was similar between the minimally invasive and conventional groups: 113 and 115 min, respectively, as was aortic clamp time: 86 and 82 min, respectively. Estimated amount of haemodilution was lower in the minimally invasive group (16.8 vs. 18.8%, *P* = 0.006). Blood transfusion frequency during surgery was less than half of conventional in the minimally invasive group (12.7 vs. 31.0%, *P* = 0.01). There were no deaths or stroke in either group during the hospital stay. Asymptomatic brain injury rate was the same for the two groups (35.2 vs. 35.2%, *P* = 1.00).

**CONCLUSIONS:**

Minimally invasive extracorporeal circulation was associated with fewer patients requiring transfusion than conventional extracorporeal circulation without an increase of asymptomatic brain injury in minimally invasive aortic valve replacement through right lateral mini-thoracotomy surgery.

## Graphical Abstract

**Figure:**
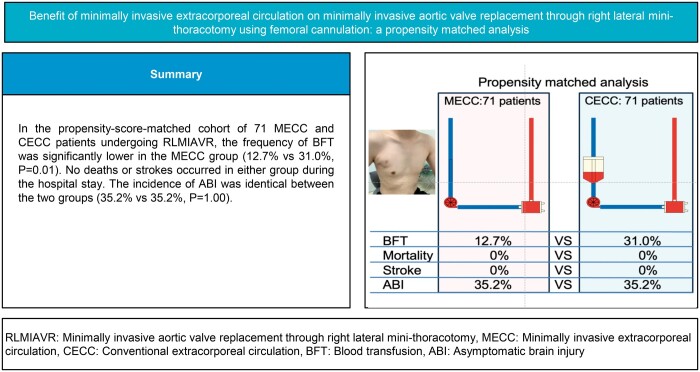

## INTRODUCTION 

Minimally invasive aortic valve replacement through right lateral mini-thoracotomy (RLMIAVR) using femoral cannulation has been demonstrated to be a safe approach. However, one drawback of RLMIAVR is its longer extracorporeal circulation (ECC) time compared to conventional aortic valve replacement (AVR) performed via full or partial median sternotomy [1–3]. Separately, surgeons now have two choices with regard to ECC: minimally invasive ECC (MECC) and conventional ECC (CECC). MECC is reported to result in better clinical outcomes than CECC, including a lower proportion of patients requiring blood transfusion [4–8]. Although there are some concerns about air handling in MECC due to the absence of a reservoir, such concerns are believed to be outweighed by the benefits of MECC and have led to its wide adoption in clinical practice.

To the best of our knowledge, there have been no reports on the outcomes of MECC use specifically in AVR through right thoracotomy. In RLMIAVR, MECC utilizes femoral venous drainage, theoretically preventing air entry from the surgical field. However, because it employs vacuum-assisted venous drainage, there is the possibility of venous collapse and consequently of the generation of microscopic air bubbles that could embolize. Therefore, the objective of this study was to evaluate the impact of MECC on blood transfusion frequency and rates of brain injury due to micro-air embolism in comparison to CECC in RLMIAVR.

## PATIENTS AND METHODS

### Patients

We retrospectively collected data from patients who underwent RLMIAVR between July 2014 and July 2021 at our institution. We reviewed postoperative magnetic resonance imaging (MRI) performed on the fifth day after surgery. This study was approved by the institutional review board of Chibanishi General Hospital (date: 2 February 2023, ID number: TGE00727-025). Written patient informed consent was obtained from all patients.

**Figure 1:**
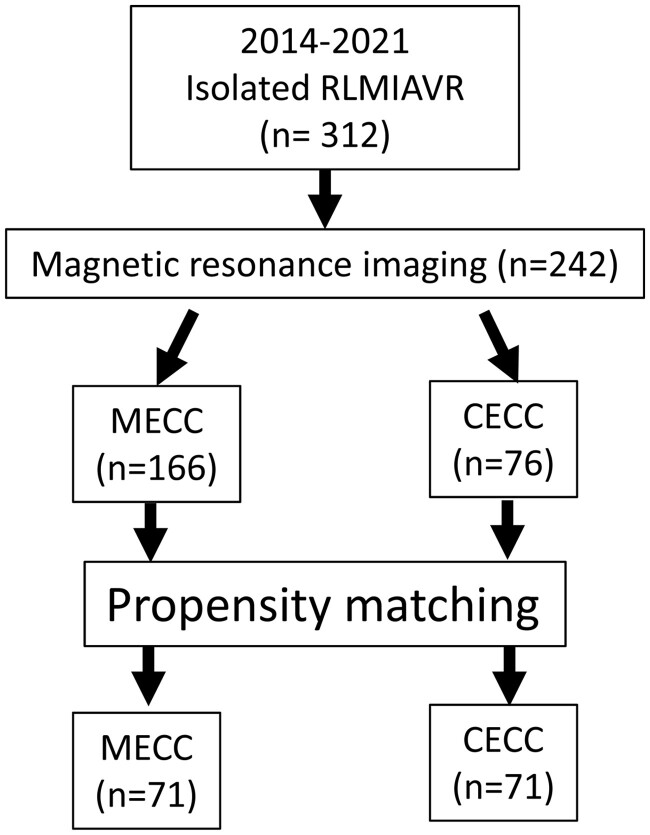
Chart of patient selection. RLMIAVR: minimally invasive aortic valve replacement through right lateral mini-thoracotomy; MECC: minimally invasive extracorporeal circulation; CECC: conventional extracorporeal circulation

We focused on postoperative outcomes, blood transfusion frequency and neurological complications, including asymptomatic brain injury (ABI) assessed by MRI. We classified patients into two groups, MECC and CECC, comprising 166 and 76 patients, respectively. Propensity matching was employed to select 71 pairs of patients for further analysis (Fig. 1).

### Surgical procedure and ECC management

We have previously detailed the RLMIAVR procedure [9, 10]. Briefly, patients are placed in a 30–40° left lateral position with a pillow under the right thorax after general anaesthesia with one lung ventilation. For anaesthesia monitoring, we track arterial pressure, pulmonary artery pressure via Swan-Ganz catheter, central venous pressure, cardiac output and transcutaneous saturations.

**Figure 2:**
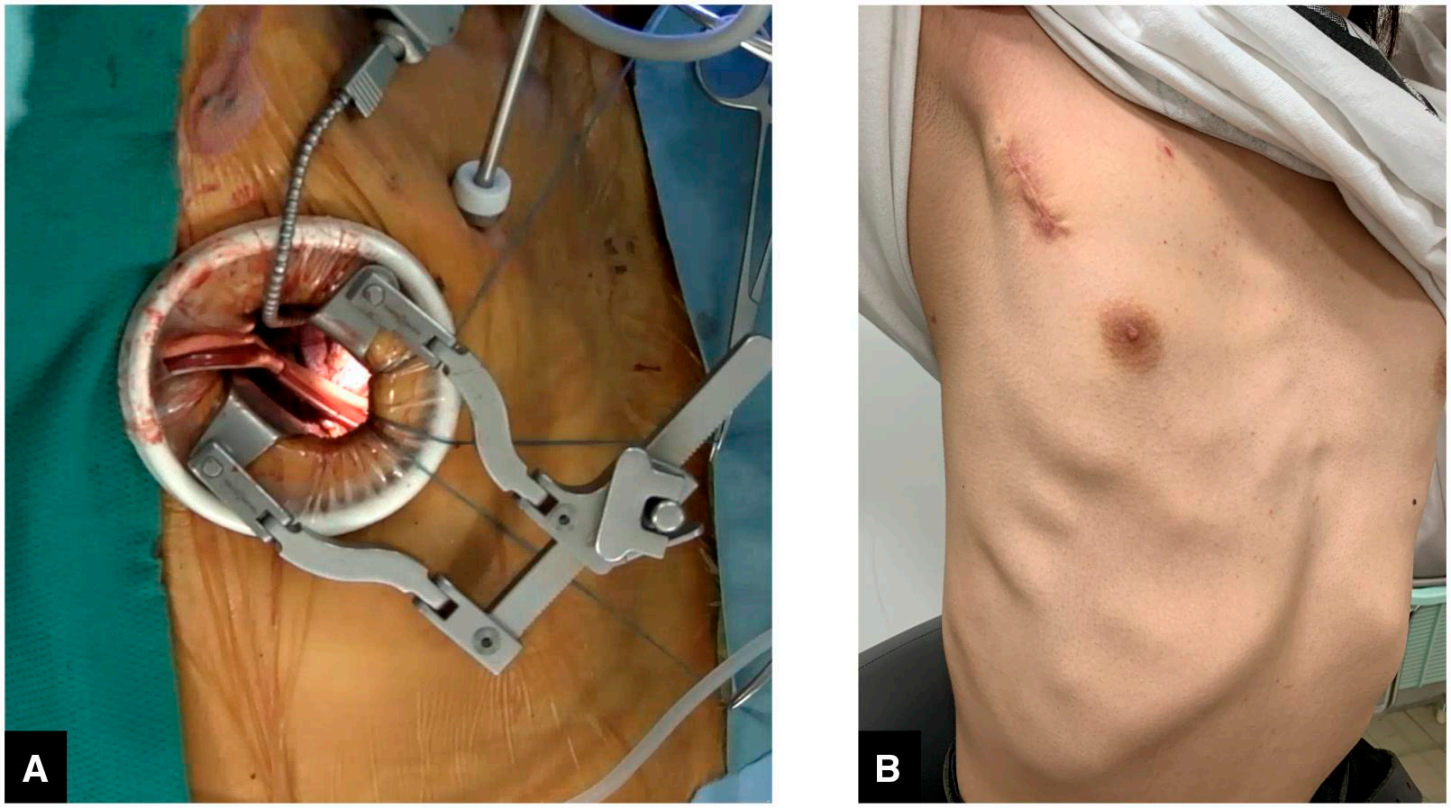
(A) Intraoperative view of the surgical field in aortic valve replacement through right lateral mini-thoracotomy. (B) Postoperative photograph of skin incision in the same patient

A 5–6 cm right lateral mini-thoracotomy is performed (Fig. 2). Before cannulation, we administer 300 units/kg of heparin. After administration, we confirm that the activated coagulation time exceeds 400 s before starting ECC. Thereafter, we measure the activated coagulation time every 30 min; if it falls below 480 s, we administer an additional 5000 units of heparin. After de-cannulation, protamine is administered at a dose of 10–15 mg for every 1000 units of heparin.

ECC is established through femoral vein cannulation using a long cannula from the femoral vein to the right atrium for venous drainage and either the femoral or axillary cannulation for systemic arterial inflow. Axillary cannulation was selected if any of the following criteria were satisfied in the aorta or an iliac artery at any point: (i) thickness of the thrombosis is >3 mm; (ii) thrombosis is more than one-third of total circumference and (iii) calcification is present in the total circumference. Femoral cannulation was selected in the remaining patients [11]. Thereafter, we measure the activated coagulation time every 30 min; if it falls below 480 s, we administer an additional 5000 units of heparin. Vacuum assistance is used for venous drainage (−40 to 60 mmHg), and perfusion is maintained between 2.2 and 2.6 l/m^2^, with the lowest blood temperature during ECC set to 32°C. Antegrade cardioplegia was delivered through aortic root cannulation, followed by selective antegrade cardioplegia. CO_2_ insufflation into the right thoracic cavity at 5 L/min and air removal in the aortic root and left ventricle is performed through vents until all air bubbles are eliminated. After de-cannulation, protamine is administered at a dose of 10–15 mg for every 1000 units of heparin.

Patients are extubated in the intensive care unit, and chest drains are removed when discharge decreases to <50 ml per 12 h. A single surgeon performed all cases in this study.

### ECC system

**Figure 3:**
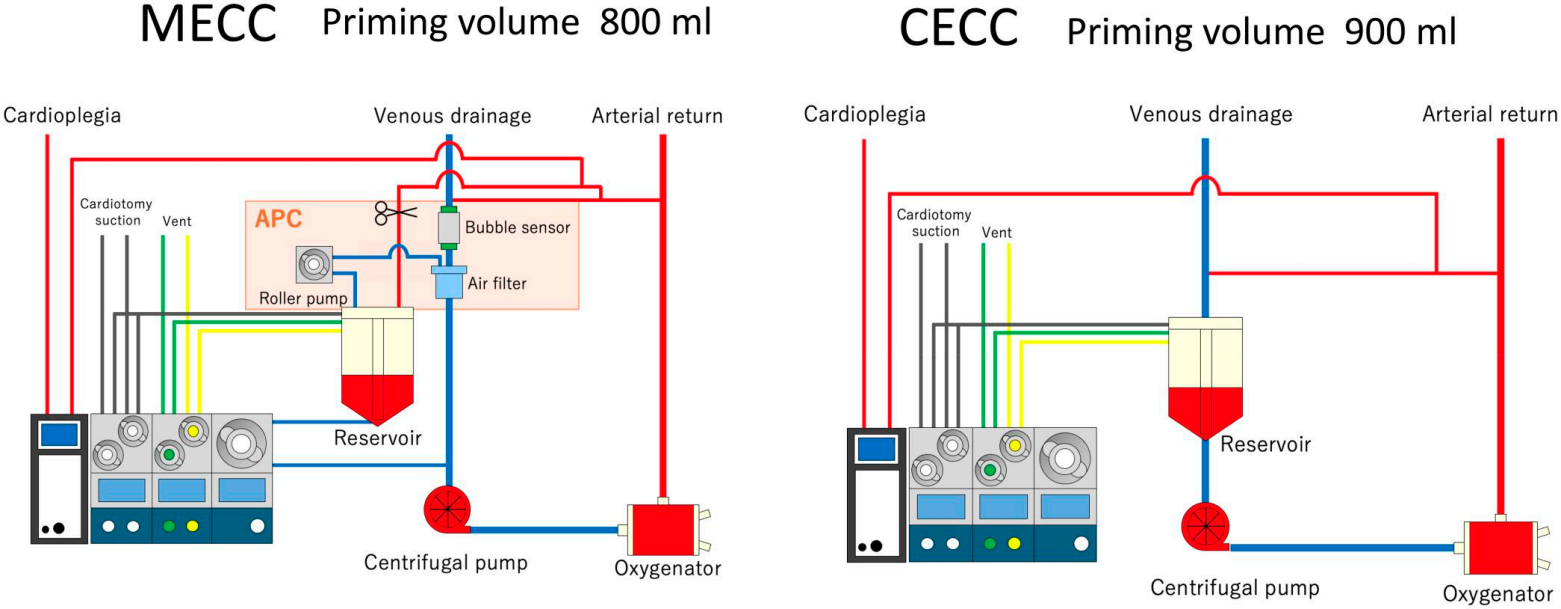
Schematic of minimally invasive extracorporeal circulation and conventional extracorporeal circulation. MECC: minimally invasive extracorporeal circulation; CECC: conventional extracorporeal circulation; APC: air purge control system

The MECC system (priming volume: 800 ml) includes a semi-closed circuit (X coating (Terumo)) with functions of oxygenation, filtration (Capiox FX advance 15/25 (Terumo)) and air purge system (Air Purge Control (LivaNova)) in a compact manner in connection with a centrifugal pump (Revolution (LivaNova)) (Fig. 3). The venous line is directly connected to a centrifugal pump without any reservoir. There is, however, a hard-shell reservoir whose only function is venting and suction connected to the circuit. This MECC system is categorized as a hybrid of type II and type IV according to the MiECTiS (Minimally Invasive Extracorporeal Technologies International Society) classification. It falls under type II because the main circuit operates without a reservoir. Simultaneously, it is classified as type IV due to the addition of a hard-shell reservoir as an auxiliary component to the circuit [12, 13].

The CECC system (priming volume: 900 ml) includes a conventional open circuit with centrifugal pump (Capiox FX advance 15/25 (Terumo)) and a hard-shell reservoir.

Since the adoption of MECC at our institution in 2014, the exclusion criteria for MECC primarily included the presence of anatomical characteristics such as possible patent foramen ovale that might risk of air entry into venous drainage and other valve diseases that might necessitate conversion to median sternotomy. Surgeon and perfusionist preferences were also taken into account. However, from 2017 to 2019, MECC was unavailable due to manufacturing issues, leading to the use of CECC in all RLMIAVR cases during that period.

Blood was transfused whenever haemoglobin level fell below 8 mg/dl. One unit of red blood cells (RBC) was 140 ml. Fresh frozen plasma (FFP) was also transfused as deemed necessary by the anaesthesiologist during surgery or by the intensivist postoperatively. One unit of FFP was 80 ml. The haemodilution rate was estimated as priming volume/(priming volume + intravascular blood volume), where intravascular blood volume (ml) was estimated as 70 times body weight (kg).

### Magnetic resonance imaging study

We previously detailed the procedure for detecting ABI using MRI [14, 15]. In short, patients underwent pre- and post-surgery MRIs with diffusion-weighted imaging (DWI) on a 3T MRI machine. Images, including axial T2-weighted and fluid attenuated inversion recovery images, trace DWI and apparent diffusion coefficient images, were reviewed by a radiologist. Neurosurgeon consultation followed lesion detection. Postoperative stroke was defined as new neurological deficits within 48 h post-surgery or thereafter. ABI was defined as new lesions detected by DWI without accompanying neurological deficits.

### Statistical analysis

We conducted propensity score matching to reduce bias in the selection of the circuit of cardiopulmonary bypass [16]. Patients from the two groups were matched in a 1:1 ratio by selecting those with the closest propensity score using the calliper matching method [14]. The calliper width was set at 20% of the standard deviation of the propensity scores. Balance in patient characteristics was assessed with the standardized mean difference (SMD). Absolute values of less than 0.1 of SMD were considered balanced. Continuous variables are expressed as median and interquartile range (IQR) and analysed with Student’s *t*-test or Mann–Whitney *U* test. Categorical variables are expressed as numbers and percentages and analysed using Fisher’s exact test. We used the following characteristics to generate the propensity score: demographic variables: age, sex, body weight, body height; clinical variables: comorbidities (including hypertension, diabetes, respiratory disease, chronic heart failure and chronic kidney disease), smoking status, European System for Cardiac Operative Risk Evaluation (EuroSCORE II), aortic stenosis, infective endocarditis, low ejection fraction (<30%), pulmonary hypertension; laboratory values: preoperative serum creatinine, haemoglobin, C-reactive protein and platelet levels. After propensity score matching, we compared the baseline characteristics between the matched groups. For continuous variables, we used paired *t*-tests to assess differences between the matched pairs. For categorical variables, we employed McNemar’s test for binary variables. All statistical analysis was conducted with SPSS Statistics, version 25.0 (SPSS Inc., Chicago, IL, USA). *P* < 0.05 was considered statistically significant.

## RESULTS

### Patient variables

In total, 312 patients underwent RLMIAVR between July 2014 and July 2021 at our institution. Among them, 242 patients underwent postoperative MRI on the fifth day after surgery. The remaining 70 patients were excluded from the study due to optout of data collection or inability to undergo MRI on the postoperative fifth day due to early discharge, the presence of pacing wires, pacemakers, oxygen or intravenous infusion equipment. Among those excluded, 48 patients underwent their surgery using MECC.

We focused on postoperative outcomes, blood transfusion rates and neurological complications, including ABI assessed by MRI. We divided patients into two groups, MECC and CECC, comprising 166 and 76 patients, respectively. Propensity matching was employed to select 71 pairs of patients for further analysis (Fig. 1).

The baseline characteristics of the patients before and after matching are summarized in Table 1.

**Table 1:** Patient characteristics in unmatched and propensity score-matched cohort

	Unmatched (*n* = 242)				Matched (*n* = 142)			
Factor	MECC (*n* = 163)	Conventional (*n* = 79)	*P*-value	SMD	MECC (*n* = 71)	Conventional (*n* = 71)	*P*-value	SMD
Age (years)	75 (67–79)	76 (70–79)	0.41	0.114	75 (70–79)	76 (70–79)	0.57	0.093
Female (%)	72 (44.2)	46 (58.2)	0.055	0.264	31 (43.7)	30 (42.3)	0.99	0.028
Body weight (kg)	57.5 (51.3–66.8)	55.2 (48.5–63.2)	0.16	0.186	56.2 (48.1–68.2)	56.3 (49.0–63.2)	0.48	0.074
Body height (cm)	160 (151–168)	154 (148–163)	0.004	0.398	156.0 (148.1–165.2)	154.4 (148.2–164.0)	0.58	0.099
CHF (%)	25 (15.3)	22 (27.9)	0.02	−0.308	19 (26.8)	17 (23.9)	0.85	0.032
CKD (%)	34 (20.9)	21 (26.6)	0.33	−0.135	19 (26.8)	16 (22.5)	0.70	0.098
DM (%)	34 (20.9)	8 (10.1)	0.046	0.300	9 (12.7)	8 (11.3)	0.99	0.043
HTN (%)	118 (72.4)	60 (76.0)	0.64	−0.081	53 (74.7)	54 (76.1)	0.99	0.033
PHTN (%)	15 (9.2)	8 (10.1)	0.82	0.031	6 (8.5)	8 (11.3)	0.79	0.095
History of stroke (%)	10 (6.1)	6 (7.8)	0.59	−0.065	4 (5.6)	6 (8.5)	0.75	0.057
Preoperative ABI (%)	8 (4.9)	5 (6.3)	0.76	−0.062	3 (4.2)	5 (5.6)	0.72	0.121
EuroSCORE II	1.32 (0.90–1.93)	1.65 (1.13–2.90)	0.002	0.251	1.35 (0.97–2.40)	1.60 (1.12–2.63)	0.97	0.007
preoperative platelet (104/µl)	19.0 (16.0–22.5)	18.6 (15.6–21.6)	0.39	0.056	18.3 (15.8–22.0)	18.6 (15.6–21.3)	0.96	0.009
preoperative haemoglobin (mg/dl)	12.9 (11.7–13.8)	12.6 (11.1–13.7)	0.09	0.228	12.7 (11.5–13.7)	12.6 (11.4–13.7)	0.46	0.037
Preoperative C-reactive protein (mg/dl)	0.05 (0.03–0.11)	0.04 (0.03–0.21)	0.41	−0.278	0.06 (0.03–0.13)	0.04 (0.03–0.17)	0.84	0.035
Femoral artery cannulation (%)	107 (65.6)	54 (68.4)	0.77	0.058	44 (62.0)	49 (69.0)	0.48	0.149

The diseases listed are comorbidities except for stroke, which is a past history of stroke.

MECC: minimally invasive extracorporeal circulation; CECC: conventional extracorporeal circulation; CHF: congestive heart failure; CKD: chronic kidney disease; pulmonary disease; DM: diabetes mellitus; HTN: hypertension; PHTN: pulmonary hypertension; ABI: asymptomatic brain injury; SMD: standardized mean difference.

### Unmatched cohort

Before matching (left half of Table 1), the MECC group differed from the CECC group in height and prevalence of congestive heart failure and diabetes mellitus. MECC patients were taller. The MECC group had a lower prevalence of history of congestive heart failure (MECC 15.3% vs. CECC 27.9%, *P* = 0.02) but a higher prevalence of diabetes mellitus (MECC: 20.9% vs. CECC 10.1%, *P* = 0.046). EuroSCORE II was significantly lower in the MECC group (MECC: 1.32 (0.90–1.93) vs. CECC: 1.65 (1.13–2.90), *P* = 0.002). History of stroke and ABI were not significantly different.

### Matched cohort

After propensity matching, the two groups (right half of Table 1) became well balanced in terms of preoperative variables, including haemoglobin level, history of congestive heart failure and diabetes and EuroSCORE II. The C-statistic of the analysis was 0.72. There was no significant difference in the choice of cannulation site between the femoral and axillary arteries.

Intraoperative and postoperative data are summarized in Table 2. In the matched group, ECC time was similar between the two groups, 113 (102–135) and 115 (100–127) min in the MECC and CECC groups, respectively. Likewise, aortic clamp time was similar, 82 (75–95) and 86 (74–101) min in the MECC and CECC groups, respectively. Mean delivery oxygen (DO_2_) during ECC was also similar between groups (MECC 315 (295–338) vs. CECC 317 (300–343) ml/min/m^2^, *P* = 0.79). Estimated degree of haemodilution in the ECC was statistically significantly lower in the MECC group (MECC: 16.8 (14.7–19.2)% vs. CECC: 18.8 (17.1–20.8)%, *P* = 0.006). Intravenous fluid volume during ECC (without cardioplegia) was not significantly different in the two group (MECC: 501 ml vs. CECC: 551 ml; P = 0.28). There was no stroke in either group. The proportion of patients requiring blood transfusion during ECC was significantly lower in the MECC group (MECC: 9/71 (12.7%) vs. CECC: 22/71 (31.0%), *P* = 0.01), below half the CECC group rate. The mean amount of RBC transfusion during ECC was significantly lower in the MECC group (MECC: 0 (0–0) unit vs. CECC: 0 (0–4) unit, *P* = 0.01). As for postoperative data, there was neither hospital death nor symptomatic stroke in either group. The ABI rate was not significantly different between the two groups (MECC: 25/71 (35.2%) vs. CECC: 25/71 (35.2%), *P* = 1.00). Transfusion rate during entire hospitalization (i.e. surgery and postoperative rate combined) was lower in MECC (MECC: 15/71 (21.1%) vs. CECC: 27/71 (38.0%), *P* = 0.04). Platelet consumption rate ((preoperative platelet count—postoperative platelet count)/preoperative platelet count) was similar between the two groups (MECC: 43.4 (35.9–52.3) vs. CECC: 39.3 (34.2–20.3)%, *P* = 0.31).

**Table 2:** Comparison of intra- and postoperative outcomes in unmatched and propensity score-matched cohort

	Unmatched (*n* = 242)			Matched (*n* = 142)		
Factor	MECC (*n* = 163)	CECC (*n* = 79)	*P*-value	MECC (*n* = 71)	CECC (*n* = 71)	*P*-value
ECC time (min)	117 (104–137)	113 (98–126)	0.14	113 (102–135)	115 (100–127)	0.66
Clamp time (min)	89 (77–103)	82 (74–95)	0.07	86 (74–101)	82 (75–95)	0.56
Estimated haemodilution rate (%)	16.8 (15.1–18.3)	18.9 (16.9–20.9)	0.001	16.8 (14.7–19.2)	18.8 (17.1–20.8)	0.006
Intravenous fluid volume during ECC (ml)	555 (454–700)	525 (418–971)	0.48	501 (410–669)	551 (406–697)	0.28
Blood transfusion frequency during ECC (%)	24 (14.7)	27 (34.2)	<0.001	9 (12.7)	22 (31.0)	0.01
FFP during ECC (unit)	0 (0–0)	0 (0–4)	0.007	0 (0–0)	0 (0–0)	0.16
RBC during ECC (unit)	0 (0–0)	0 (0–4)	<0.001	0 (0–0)	0 (0–4)	0.01
Blood transfusion frequency during hospitalization (%)	33 (20.3)	32 (40.5)	0.001	15 (21.1)	27 (38.0)	0.04
Postoperative FFP (unit)	0 (0–0)	0 (0–0)	0.22	0 (0–0)	0 (0–0)	0.27
Postoperative RBC (unit)	0 (0–0)	0 (0–0)	0.81	0 (0–0)	0 (0–0)	0.91
Hospital death (%)	0 (0)	0 (0)	–	0 (0)	0 (0)	–
Maximum CPK (U/L)	808 (543–1287)	656 (488–1007)	0.43	707 (504–1167)	659 (488–1004)	0.79
Maximum lactate (mmol/L)	4.1 (2.8–5.6)	3.7 (2.4–5.2)	0.13	3.9 (2.8–5.6)	3.7 (2.3–4.8)	0.14
DO_2_ (ml/min)	312.9 (293–339.1)	322.6 (304.2–345.9)	0.06	315.4 (294.9–338.4)	317.0 (300.3–343.0)	0.79
Platelet consumption rate (%)	41.8 (36.3–50.3)	40.8 (34.2–51.8)	0.18	43.4 (35.9–52.3)	39.3 (34.2–50.3)	0.31
Postoperative cerebral infarction (%)	0 (0)	0 (0)	–	0 (0)	0 (0)	–
Postoperative ABI on MRI (%)	58 (35.6)	30 (38.5)	0.67	25 (35.2)	25 (35.2)	1.00
Acute kidney injury (%)	16 (9.8)	4 (5.1)	0.32	7 (9.9)	3 (4.2)	0.34
Intubation time (h)	7 (5–10)	7 (6–9)	0.88	7 (5–11)	7 (6–9)	0.26
Chest drain output (ml)	140 (70–270)	200 (120–410)	0.004	145 (72–270)	190 (100–410)	0.03
Hospital stay (days)	8 (7–10)	9 (7–12)	0.049	9 (7–12)	9 (7–12)	0.36

Dilution rate was calculated by the following formula: priming volume/(priming volume + intravascular blood volume).

MECC: minimally invasive extracorporeal circulation; CECC: conventional extracorporeal circulation; ECC: extracorporeal circulation; FFP: fresh frozen plasma; RBC: red blood cells; BTF: blood transfusion; Postop.: postoperative; CPK: creatine phosphokinase; DO_2_: oxygen delivery; ABI: asymptomatic brain injury; MRI: magnetic resonance imaging.

## DISCUSSION

The main findings of the propensity-matched study were: (1) the blood transfusion rate was lower in the MECC group than in the CECC group both during the RLMIAVR surgery and postoperatively; (2) there was no hospital death or stroke in either group; and (3) there was no significant difference in terms of incidence of ABI.

This study has three major strengths. First, this is the first report of MECC exclusively in RLMIAVR using femoral venous vacuum drainage. Second, this study compared frequency of blood transfusion between MECC and CECC for a single surgical procedure, RLMIAVR [9]. This minimized the variance in amount of bleeding from the surgical field that comes from type of surgery, leading us to more easily attribute the difference in transfusion rates to the type of ECC employed. Third, all of the patients in this study underwent MRI, allowing us to identify ABI. Previous research on MECC and neurological complications has lacked MRI examination, so only symptomatic brain lesions could be assessed, leaving doubt as to the relative safety of MECC vs. CECC [4, 17].

In this study, we found that the proportion of patients requiring blood transfusion both during and after the surgical procedure was lower in the MECC group compared to the CECC group. The reasons may be, firstly, haemodilution rate is lower in an MECC circuit. MECC has a 100 ml less priming volume, a relatively high amount in relation to the smaller framed Japanese subjects in this study, and the high transfusion threshold we employed of haemoglobin <8 mg/dl. Second, in CECC, when the blood volume in the reservoir approaches depletion, perfusionists tend to add intravenous fluid to prevent air introduction into the arterial line. In contrast, this is not necessary in MECC, as the circuit lacks a main reservoir. Based on this, we speculated that less intravenous fluid might have been required in MECC. However, in our study, the amount of intravenous fluid administered during ECC was only 50 ml (10%) more in the CECC group (MECC: 501 ml vs. CECC: 551 ml; *P* = 0.28), with no statistically significant difference, though there was a tendency towards higher fluid volumes in MECC. This slight difference could have potentially contributed to the minor variation in haemodilution observed between the two systems. Third, there is less blood–air contact in MECC, which leads to less blood loss via coagulation and inflammatory responses [18, 19].

Our study was unique in its focus on RLMIAVR. A meta-analysis of 42 MECC vs. CECC comparative studies entirely consists of coronary artery bypass grafting (CABG) and/or AVR surgery studies [4]. The analysis found that MECC procedures significantly reduced the transfusion volume of RBCs and also of other blood products. Yilmaz *et al.* studied combined CABG and AVR surgery patients and found that MECC reduced the proportion of patients needing blood products, both intra- and postoperatively [6]. Ellam *et al.* studied CABG patients exclusively and found a reduction of patients needing RBC transfusions with MECC only during surgery [7]. Since RLMIAVR does not involve cutting the sternum or ribs, there is less bleeding whether intra- or postoperatively, as can be seen from the very low postoperative chest drain output in both our groups (MECC 145, CECC 190 ml). In contrast, CABG and AVR are usually performed through median sternotomy, which is associated with greater bleeding and wider variability in volume of bleeding. This variability may make it harder to tease out differences between MECC and CECC.

Neither the MECC nor CECC group showed any stroke occurrence, and ABI incidence rates were comparable. The primary distinction between MECC and CECC is the absence of a reservoir in the former’s circuit. Without a reservoir to trap air, venous air could potentially pass into the arterial line, causing cerebral infarction. This risk is a concern in open-heart surgery using MECC [20]. Basciani *et al.* conducted a randomized trial comparing MECC and CECC in AVR patients, reporting a significantly higher incidence of high-intensity transient signals (with MECC, measured via transcranial Doppler ultrasound [21]. They recommended oxygenators with integrated arterial line filters for MECC. However, in RLMIAVR, the femoral cannula insertion site in MECC is distant from the right atrium, reducing air entry risk. Additionally, the MECC circuit in this study included an arterial line filter and an Air Purge Control system, which aspirates air and blood into a reservoir for venting when air is detected, mitigating embolization risks [19].

ABI incidence was approximately 35% in both groups, aligning with the literature: 29% after median sternotomy, 47% after valve surgery and 38% after AVR [22]. Micro-air embolization is considered a possible cause of ABI and concerns specific to vacuum drainage in RLMIAVR include venous collapse (venous wall adhering to the cannula) resulting in microscopic air bubble generation from the overly negative pressure. While this phenomenon can occur with both MECC and CECC, it is only of concern with MECC because of the lack of a reservoir in the main circuit. However, the similarity in ABI incidence in our study demonstrates that at least in RLMIAVR, the use of MECC does not increase micro-air embolization. To reiterate, ours is the first study to measure the incidence of ABI in MECC in RLMIAVR.

### Study limitations

Our study was a non-randomized retrospective study of MECC vs. CECC. A prospective design with randomization would be preferable. Second, this single-centre study, with a modest patient sample, involved surgeries performed by one surgeon and team. Rates of complications and transfusion requirements may differ at other institutions. However, our study design also allowed for a more reliable comparison of the one ECC method over the other. Third, MRI timing limited precise ABI occurrence analysis. Fourth, the small patient sample size and the relatively high transfusion threshold (haemoglobin level <8 mg/dl) may affect the transfusion rates. Lastly, there was a selection bias regarding MECC or CECC, influenced by the preferences of the surgeon and perfusionist and anatomical feasibility such as the potential for air entry or conversion to median sternotomy.

## CONCLUSION

In patients undergoing RLMIAVR, MECC was associated with a smaller number of patients requiring blood transfusion than CECC and did not lead to an increase of ABI as assessed by MRI.

## Data Availability

The data underlying this article will be shared on reasonable request to the corresponding author.

## ABBREVIATIONS

ABI: Asymptomatic brain injury
AVR: Aortic valve replacement
CECC: Conventional ECC
DWI: Diffusion-weighted imaging
ECC: Extracorporeal circulation
MECC: Minimally invasive ECC
MRI: Magnetic resonance imaging
RBC: Red blood cells
RLMIAVR: Minimally invasive aortic valve replacement through right lateral mini-thoracotomy
